# Dioxidomolybdenum(VI) complex featuring a 2,4-di­fluoro-substituted amine bis­(phenolate) ligand

**DOI:** 10.1107/S2414314621005162

**Published:** 2021-05-21

**Authors:** Christina L. Bowen, Bradley M. Wile

**Affiliations:** a The School of Science, Technology, and Mathematics, Ohio Northern University, 525 S. Main Street, Ada, OH 45810, USA; University of Kentucky, USA

**Keywords:** crystal structure, molybdenum, oxo, Moco

## Abstract

Synthetic complexes containing a *cis*-[MoO_2_]^2+^ core are well-established models for the molybdenum co-factor (Moco). Here we report the crystal structure of such a model complex bearing a tetra­dentate amine bis­(phenolate) ligand with fluorine substituents on the phenolate rings, namely, [2,2′-({[2-(di­methyl­amino)­eth­yl]aza­nedi­yl}bis­(methyl­ene))bis­(4,6-di­fluoro­phenolato)]dioxidomolybden­um(VI)), [Mo(C_18_H_18_F_4_N_2_O_2_)O_2_]. Distortion from idealized octa­hedral symmetry about the Mo center is evident in the large O=Mo=O angle [108.54 (4)°] and the small N–Mo–O_phenolate_ angles [79.79 (4), 81.21 (3), 77.83 (3), and 84.59 (3)°]. The dihedral angle between the phenolate rings is 60.06 (4)°, and π–π stacking is observed between aromatic rings related by inversion (1 − *x*, 1 − *y*, 1 − *z*). The lower data-collection temperature of 150 K *vs* room-temperature data collection reported previously [KOWXIF; Cao *et al.* (2014 ▸). *Transit. Met. Chem.* **39**, 933–937] and larger 2*θ* range for data collection (5.8–66.6° *versus* 6–54.96°) led to a structure with lower *R*_1_ and *ωR*_2_ values (0.019 and 0.049 *vs* 0.0310 and 0.0566 for KOWXIF). Comparison of the metrical parameters with KOWXIF suggests that this dataset offers a more realistic depiction of bonding within the Mo^VI^=O moiety.

## Abstract 2

An updated structure is reported for a bis­(oxo) Mo^VI^ complex that is modeled after the molybdenum cofactor. An analysis of lengths and angles suggests that this dataset offers a more accurate depiction of bonding for the Mo^VI^=O moiety.

## Graphical abstract

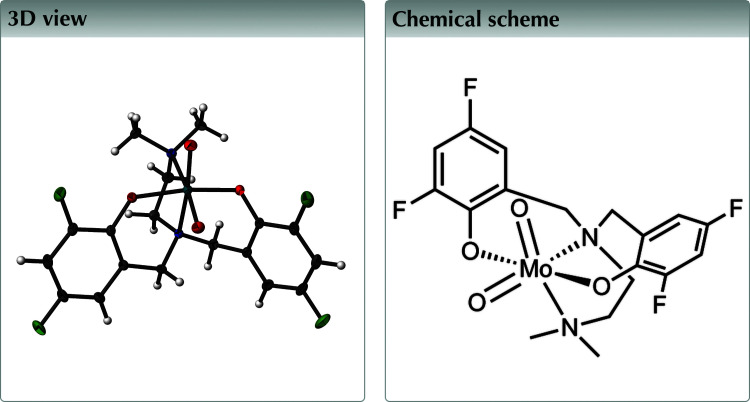

## Structure description

Molybdenum-containing metalloenzymes are abundant and serve as excellent motivation for biomimetic catalyst development. The relevance of xanthine oxidase, DMSO reductase, sulfite oxidase to oxygen atom transfer and proton-coupled electron-transfer reactions have driven inter­est in related mononuclear Mo complexes for generating H_2_ or expanding opportunities for storage of energy generated by increasingly efficient solar cells. The native enzymes contain a molybdenum cofactor (Moco) in which the Mo^VI^=O moiety is supported by di­thiol­ene-containing molybdopterin ligands (Kisker *et al.*, 1997 ▸). A robust molybdenum–oxo complex bearing a penta­dentate pyridyl ligand was notably shown to catalytically generate H_2_ from water at a low overpotential (Karunadasa *et al.*, 2010 ▸). Related molybdenum–oxo complexes featuring an amine bis­(phenolate) moiety have been similarly shown to promote H_2_ generation (Cao *et al.*, 2014 ▸) and oxygen atom transfer (Maurya *et al.*, 2016 ▸). Insight into the structural features that enable such activity at the Mo=O moiety are thus an important component of iterating the design of these species for use as sustainable aqueous catalysts.

**Figure 1:**
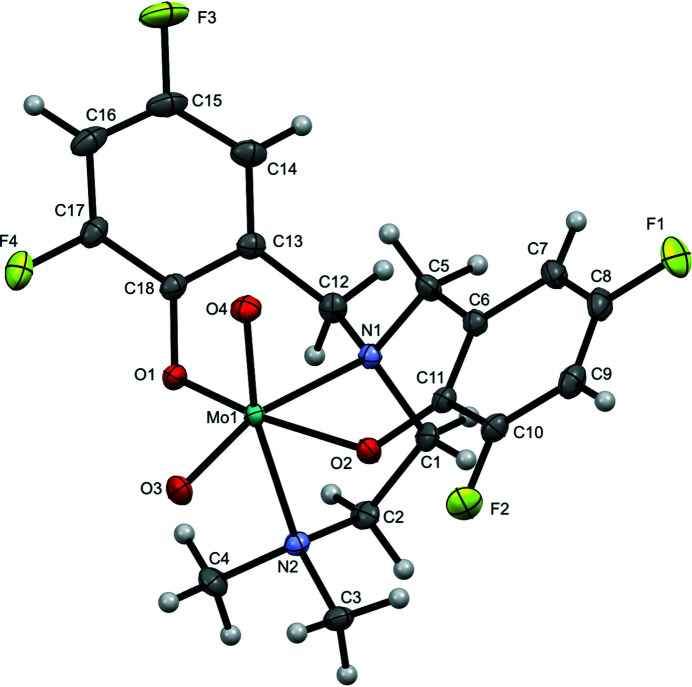
Mol­ecular structure of **2** with 50% displacement ellipsoids and the numbering scheme for non-H atoms.

The Mo complex reported here (**2**, Fig. 1 ▸) is chemically identical to that reported by Cao *et al.* (2014 ▸, KOWXIF). The lower collection temperature (150 K *versus* room temperature in KOWXIF) and larger 2*θ* range for data collection (5.8–66.6° *versus* 6–54.96° in the previous report) led to a structure solution with lower *R*_1_ and *ωR*_2_ values (0.019 and 0.049 *versus* 0.0310 and 0.0566 in KOWXIF). Slight differences in the bond lengths for the compound in these structures warrant further comment and may be of inter­est from a mechanistic perspective. For example, it is generally accepted that an Mo=O bond in the *cis*-[MoO_2_]^2+^ core of DMSO reductase model compounds is formally strengthened (consistent with Mo≡O) during oxygen atom transfer (Enemark, *et al.*, 2004 ▸).

Both structures have *P* 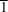 space-group symmetry, though *a*, *c*, β, and γ were different by ±3 s.u. While the Mo—O(phenolate) and Mo—N bonds in this structure are nearly identical to those reported by Cao *et al.*, the Mo=O bond lengths reported here are notably longer than those in KOWXIF and are in line with expectations for related Mo^VI^–oxo species (Enemark, *et al.*, 2004 ▸). However, these differences in bond length are within the accuracy limits for light atoms imposed by the spherical atom scattering factor approximation (*e.g.* Dawson, 1964 ▸). Relevant lengths and angles for both are summarized in Table 1 ▸. Differences in the metrical parameters for these structures suggest that the model presented here gives a better representation of the bonding for **2**, when compared with other Mo^VI^ oxo species.

**Figure 2:**
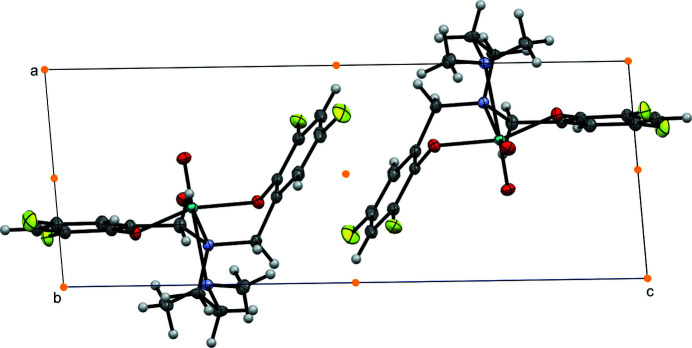
Packing plot (viewed along *b*) showing π–π stacking of fluorinated (F3, F4) phenolate rings related by inversion. Orange dots represent centers of inversion.

No hydrogen bonding was observed, though short contacts exist between inversion-related mol­ecules contained in the unit cell. The orientation of one phenolate ring brings the *ortho* carbons C16 and C18^i^ [symmetry code: (i) 1 − *x*, 1 − *y*, 1 − *z*] in close proximity [3.2807 (15) Å, *i.e.* ∼0.12 Å closer than the sum of the vdW radii]. Close contact is noted for the *para* F3 and proximal *meta* C16^ii^ [symmetry code: (ii) 2 − *x*, 2 − *y*, 1 − *z*] of an adjacent inversion-related mol­ecule [3.1622 (14) Å, *i.e.* ∼0.01 Å closer than the sum of the vdW radii]. This marginally short contact is consistent with π–π stacking between the phenolate rings related by the inversion center; this inter­action is shown in Fig. 2 ▸. A similar, though much more pronounced contact is noted between *ortho* F2 and the amine methyl group, C3^iii^ [symmetry code: (iii) −*x*, −*y*, −*z*] [2.9229 (13) Å, ∼0.247 Å closer than the sum of the vdW radii]. Each Mo^VI^=O moiety lies above planes defined by the amine bis­(phenolate) ligand and the other oxo [0.3037 (4) Å above the plane defined by O1, O2, O4, and N2; 0.2696 (4) Å above the plane defined by O1, O2, O3, and N1]. This distortion from an ideal octa­hedral geometry is consistent with related Mo^VI^ oxo species, and is evident in the large O3—Mo1—O4 bond angle [108.54 (4)°]. The dihedral between aromatic rings was found to be 60.06 (4)°. The torsion angle along the di­amine N1—C1—C2—N2 [−55.84 (11)°] is consistent with the *syn* conformation of amine donors within the unstrained five-membered ring formed upon chelation.

## Synthesis and crystallization

**Figure 3:**
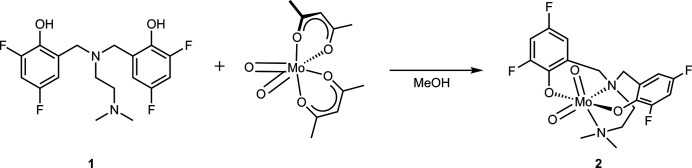
Reaction scheme.

The ligand H_2_ONNO^F^ (**1**) was prepared by the method reported previously (Graziano *et al.*, 2019 ▸). The Mo complex (**2**) was prepared using a modified version of the method reported by Lehtonen & Sillanpää (2005 ▸). The reaction scheme is shown in Fig. 3 ▸. MoO_2_(acac)_2_ (0.330 g, 1.01 mmol) and the ligand H_2_ONNO^F^ (**1**; 0.373 g, 1.00 mmol) were combined in a 20 ml scintillation vial with a PTFE-coated stir bar and suspended in 10 ml of anhydrous methanol. The reaction mixture was left to stir for 4 h at 295 K, at which time solvent and other volatiles were removed *in vacuo* to yield a yellow solid (0.500 g, 1.00 mmol, >99%). The product was purified by column chromatography on silica using an increasing linear gradient of di­chloro­methane in acetone as the eluent. After removing the solvent and other volatiles, single crystals suitable for diffraction studies were obtained by slow evaporation from a concentrated solution of acetone. Characterization data for this compound match those previously reported by Cao *et al.* (2014 ▸). M.p. = 463–467 K.

## Refinement

Crystal data, data collection and structure refinement details are summarized in Table 2 ▸. No disorder or solvent were present.

## Supplementary Material

Crystal structure: contains datablock(s) I, global. DOI: 10.1107/S2414314621005162/pk4033sup1.cif

Structure factors: contains datablock(s) I. DOI: 10.1107/S2414314621005162/pk4033Isup2.hkl

CCDC reference: 2083619

Additional supporting information:  crystallographic information; 3D view; checkCIF report

## Figures and Tables

**Table 1:** Comparison of lengths and angles between this work and previous report

	This work (CLB-1–87)	KOWXIF (Cao *et al.*, 2014 ▸)	
Mo—O1	1.9788 (8)	1.976 (3)	
Mo—O2	1.9287 (8)	1.919 (3)	
Mo—O3	1.7062 (8)	1.693 (2)	difference greater than ±3 s.u.
Mo—O4	1.7123 (7)	1.700 (3)	difference greater than ±3 s.u.
Mo—N1	2.4008 (8)	2.395 (3)	
Mo—N2	2.4117 (9)	2.412 (3)	
			
O1—Mo1—O3	98.19 (4)	98.6 (1)	
O1—Mo1—O4	93.41 (4)	93.4 (1)	
O1—Mo1—N1	79.79 (3)	79.7 (1)	
O1—Mo1—N2	84.59 (3)	84.5 (1)	
O2—Mo1—O3	99.96 (4)	99.9 (1)	
O2—Mo1—O4	95.43 (4)	95.8 (1)	
O2—Mo1—N1	77.83 (3)	77.5 (1)	
O2—Mo1—N2	81.21 (3)	81.0 (1)	
O3—Mo1—O4	108.54 (4)	108.2 (1)	
N1—Mo1—N2	73.84 (3)	73.8 (1)	
			
N1—C1—C2—N2	−55.84 (11)	56.4 (4)	

**Table 2:** Experimental details

Crystal data	
Chemical formula	[Mo(C_18_H_18_F_4_N_2_O_2_)O_2_]
*M*_r_	498.28
Crystal system, space group	Triclinic, *P* 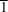
Temperature (K)	150
*a*, *b*, *c* (Å)	7.3179 (4), 8.0093 (4), 17.5057 (9)
α, β, γ (°)	91.8513 (18), 92.9102 (18), 116.6842 (16)
*V* (Å^3^)	913.82 (8)
*Z*	2
Radiation type	Mo *K*α
μ (mm^−1^)	0.79
Crystal size (mm)	0.26 × 0.24 × 0.15

Data collection	
Diffractometer	Bruker AXS D8 Quest CMOS diffractometer
Absorption correction	Multi-scan (*SADABS*; Krause *et al.*, 2015 ▸)
*T*_min_, *T*_max_	0.702, 0.747
No. of measured, independent and observed [*I* > 2σ(*I*)] reflections	47859, 7013, 6541
*R*_int_	0.027
(sin θ/λ)_max_ (Å^−1^)	0.772

Refinement	
*R*[*F* ^2^ > 2σ(*F* ^2^)], *wR*(*F* ^2^), *S*	0.019, 0.049, 1.12
No. of reflections	7013
No. of parameters	266
H-atom treatment	H-atom parameters constrained
Δρ_max_, Δρ_min_ (e Å^−3^)	0.92, −0.48

Computer programs: *APEX3* and *SAINT* (Bruker, 2018 ▸), *SHELXT* (Sheldrick, 2015*a* ▸), *SHELXL2018/3* (Sheldrick, 2015*b* ▸), *ShelXle* (Hübschle *et al.*, 2011 ▸) and *Mercury* (Macrae *et al.*, 2020 ▸).

## full crystallographic data

### Crystal data

**Table:**

[Mo(C_18_H_18_F_4_N_2_O_2_)O_2_]	*Z* = 2
*M**_r_* = 498.28	*F*(000) = 500
Triclinic, *P*1	*D*_x_ = 1.811 Mg m^−^^3^
*a* = 7.3179 (4) Å	Mo *K*α radiation, λ = 0.71073 Å
*b* = 8.0093 (4) Å	Cell parameters from 9173 reflections
*c* = 17.5057 (9) Å	θ = 2.3–33.2°
α = 91.8513 (18)°	µ = 0.79 mm^−^^1^
β = 92.9102 (18)°	*T* = 150 K
γ = 116.6842 (16)°	Block, orange
*V* = 913.82 (8) Å^3^	0.26 × 0.24 × 0.15 mm

### Data collection

**Table:**

Bruker AXS D8 Quest CMOS diffractometer	7013 independent reflections
Radiation source: fine focus sealed tube X-ray source	6541 reflections with *I* > 2σ(*I*)
Triumph curved graphite crystal monochromator	*R*_int_ = 0.027
Detector resolution: 10.4167 pixels mm^-1^	θ_max_ = 33.3°, θ_min_ = 2.9°
ω and phi scans	*h* = −11→11
Absorption correction: multi-scan (*SADABS*; Krause *et al.*, 2015)	*k* = −12→12
*T*_min_ = 0.702, *T*_max_ = 0.747	*l* = −26→25
47859 measured reflections	

### Refinement

**Table:**

Refinement on *F*^2^	Secondary atom site location: difference Fourier map
Least-squares matrix: full	Hydrogen site location: inferred from neighbouring sites
*R*[*F*^2^ > 2σ(*F*^2^)] = 0.019	H-atom parameters constrained
*wR*(*F*^2^) = 0.049	*w* = 1/[σ^2^(*F*_o_^2^) + (0.0194*P*)^2^ + 0.4598*P*] where *P* = (*F*_o_^2^ + 2*F*_c_^2^)/3
*S* = 1.12	(Δ/σ)_max_ = 0.001
7013 reflections	Δρ_max_ = 0.92 e Å^−^^3^
266 parameters	Δρ_min_ = −0.48 e Å^−^^3^
0 restraints	Extinction correction: *SHELXL2018/3* (Sheldrick 2015b), Fc^*^=kFc[1+0.001xFc^2^λ^3^/sin(2θ)]^-1/4^
Primary atom site location: structure-invariant direct methods	Extinction coefficient: 0.0171 (8)

### Special details

**Table:**

Geometry. All esds (except the esd in the dihedral angle between two l.s. planes) are estimated using the full covariance matrix. The cell esds are taken into account individually in the estimation of esds in distances, angles and torsion angles; correlations between esds in cell parameters are only used when they are defined by crystal symmetry. An approximate (isotropic) treatment of cell esds is used for estimating esds involving l.s. planes.

### Fractional atomic coordinates and isotropic or equivalent isotropic displacement parameters (Å^2^)

**Table:**

	*x*	*y*	*z*	*U*_iso_*/*U*_eq_	
Mo1	0.35403 (2)	0.27852 (2)	0.23176 (2)	0.01088 (3)	
F1	0.30483 (14)	0.85179 (12)	−0.04843 (5)	0.03105 (18)	
F2	0.23146 (13)	0.24459 (11)	−0.02051 (4)	0.02510 (15)	
F3	0.77983 (14)	0.98402 (12)	0.50141 (5)	0.0357 (2)	
F4	0.74459 (12)	0.40003 (12)	0.42642 (5)	0.02571 (15)	
O2	0.24235 (12)	0.28426 (11)	0.13025 (4)	0.01475 (13)	
O1	0.37637 (12)	0.30006 (11)	0.34515 (4)	0.01499 (13)	
O3	0.39279 (13)	0.08489 (11)	0.21952 (5)	0.01831 (15)	
O4	0.58252 (12)	0.47327 (11)	0.22506 (5)	0.01743 (14)	
N1	0.18199 (13)	0.46786 (12)	0.25408 (5)	0.01204 (14)	
N2	0.00004 (13)	0.06353 (12)	0.24545 (5)	0.01375 (15)	
C1	−0.03936 (15)	0.35240 (15)	0.22876 (6)	0.01565 (18)	
H1A	−0.055488	0.333606	0.172183	0.019*	
H1B	−0.118429	0.419086	0.244487	0.019*	
C2	−0.12266 (16)	0.16343 (15)	0.26393 (7)	0.01739 (18)	
H2A	−0.119764	0.181957	0.320249	0.021*	
H2B	−0.267036	0.086095	0.244251	0.021*	
C3	−0.08879 (17)	−0.05342 (15)	0.17262 (6)	0.01864 (19)	
H3A	−0.099801	0.024782	0.132488	0.028*	
H3B	−0.225312	−0.153590	0.180273	0.028*	
H3C	0.000294	−0.108430	0.157166	0.028*	
C4	−0.01342 (18)	−0.06387 (16)	0.30686 (7)	0.0197 (2)	
H4A	−0.157319	−0.152867	0.311224	0.029*	
H4B	0.043768	0.009500	0.355647	0.029*	
H4C	0.064405	−0.132458	0.294289	0.029*	
C5	0.27534 (17)	0.63562 (14)	0.20820 (6)	0.01565 (18)	
H5A	0.419181	0.712021	0.228692	0.019*	
H5B	0.201603	0.711773	0.216119	0.019*	
C6	0.27467 (16)	0.59822 (14)	0.12285 (6)	0.01432 (17)	
C7	0.29093 (17)	0.74157 (16)	0.07532 (6)	0.01792 (19)	
H7	0.303492	0.856995	0.096826	0.021*	
C8	0.28844 (18)	0.71284 (17)	−0.00284 (7)	0.0202 (2)	
C9	0.26776 (18)	0.54719 (17)	−0.03773 (6)	0.0203 (2)	
H9	0.265023	0.529975	−0.091799	0.024*	
C10	0.25135 (17)	0.40861 (16)	0.01002 (6)	0.01692 (18)	
C11	0.25771 (15)	0.43022 (14)	0.08992 (6)	0.01363 (16)	
C12	0.19506 (17)	0.53698 (15)	0.33577 (6)	0.01572 (18)	
H12A	0.159066	0.642284	0.336701	0.019*	
H12B	0.091704	0.435217	0.363450	0.019*	
C13	0.40118 (16)	0.60117 (15)	0.37784 (6)	0.01528 (17)	
C14	0.50261 (19)	0.77546 (16)	0.41804 (6)	0.0204 (2)	
H14	0.449901	0.864217	0.415639	0.024*	
C15	0.6818 (2)	0.81511 (17)	0.46142 (7)	0.0238 (2)	
C16	0.76763 (18)	0.69352 (18)	0.46549 (7)	0.0229 (2)	
H16	0.892175	0.725565	0.495342	0.028*	
C17	0.66479 (17)	0.52275 (16)	0.42429 (6)	0.01820 (19)	
C18	0.47924 (16)	0.47139 (14)	0.38146 (6)	0.01454 (17)	

### Atomic displacement parameters (Å^2^)

**Table:**

	*U*^11^	*U*^22^	*U*^33^	*U*^12^	*U*^13^	*U*^23^
Mo1	0.01022 (4)	0.01143 (4)	0.01228 (4)	0.00609 (3)	0.00069 (3)	0.00012 (3)
F1	0.0408 (5)	0.0298 (4)	0.0230 (4)	0.0155 (4)	0.0016 (3)	0.0148 (3)
F2	0.0352 (4)	0.0255 (4)	0.0153 (3)	0.0151 (3)	−0.0014 (3)	−0.0060 (3)
F3	0.0359 (5)	0.0233 (4)	0.0305 (4)	−0.0005 (3)	−0.0042 (3)	−0.0120 (3)
F4	0.0204 (3)	0.0338 (4)	0.0257 (4)	0.0149 (3)	−0.0020 (3)	0.0034 (3)
O2	0.0190 (3)	0.0142 (3)	0.0122 (3)	0.0086 (3)	0.0002 (3)	0.0003 (2)
O1	0.0168 (3)	0.0141 (3)	0.0131 (3)	0.0064 (3)	−0.0015 (3)	0.0004 (2)
O3	0.0197 (4)	0.0168 (3)	0.0226 (4)	0.0121 (3)	0.0000 (3)	−0.0005 (3)
O4	0.0132 (3)	0.0168 (3)	0.0207 (4)	0.0052 (3)	0.0030 (3)	0.0011 (3)
N1	0.0128 (3)	0.0130 (3)	0.0117 (3)	0.0070 (3)	0.0008 (3)	0.0003 (3)
N2	0.0130 (4)	0.0134 (4)	0.0146 (4)	0.0056 (3)	0.0011 (3)	0.0011 (3)
C1	0.0116 (4)	0.0176 (4)	0.0199 (5)	0.0088 (3)	−0.0005 (3)	0.0006 (3)
C2	0.0114 (4)	0.0181 (4)	0.0228 (5)	0.0066 (3)	0.0032 (3)	0.0013 (4)
C3	0.0171 (4)	0.0155 (4)	0.0183 (5)	0.0035 (4)	−0.0019 (4)	−0.0024 (4)
C4	0.0220 (5)	0.0165 (4)	0.0196 (5)	0.0073 (4)	0.0040 (4)	0.0067 (4)
C5	0.0218 (5)	0.0123 (4)	0.0142 (4)	0.0088 (4)	0.0018 (3)	0.0012 (3)
C6	0.0147 (4)	0.0148 (4)	0.0136 (4)	0.0067 (3)	0.0007 (3)	0.0019 (3)
C7	0.0195 (5)	0.0169 (4)	0.0180 (5)	0.0086 (4)	0.0009 (4)	0.0047 (4)
C8	0.0194 (5)	0.0226 (5)	0.0178 (5)	0.0084 (4)	0.0009 (4)	0.0086 (4)
C9	0.0187 (5)	0.0276 (5)	0.0132 (4)	0.0092 (4)	0.0004 (4)	0.0041 (4)
C10	0.0165 (4)	0.0203 (5)	0.0133 (4)	0.0080 (4)	−0.0004 (3)	−0.0012 (3)
C11	0.0130 (4)	0.0159 (4)	0.0119 (4)	0.0065 (3)	0.0002 (3)	0.0009 (3)
C12	0.0183 (4)	0.0188 (4)	0.0129 (4)	0.0108 (4)	0.0025 (3)	−0.0004 (3)
C13	0.0177 (4)	0.0154 (4)	0.0111 (4)	0.0060 (3)	0.0020 (3)	0.0001 (3)
C14	0.0250 (5)	0.0170 (5)	0.0155 (5)	0.0062 (4)	0.0032 (4)	−0.0019 (4)
C15	0.0247 (5)	0.0192 (5)	0.0152 (5)	−0.0006 (4)	0.0014 (4)	−0.0041 (4)
C16	0.0177 (5)	0.0267 (5)	0.0138 (5)	0.0008 (4)	−0.0008 (4)	0.0007 (4)
C17	0.0149 (4)	0.0230 (5)	0.0137 (4)	0.0059 (4)	0.0006 (3)	0.0030 (4)
C18	0.0146 (4)	0.0158 (4)	0.0107 (4)	0.0046 (3)	0.0007 (3)	0.0011 (3)

### Geometric parameters (Å, º)

**Table:**

Mo1—O3	1.7062 (8)	C4—H4A	0.9800
Mo1—O4	1.7123 (8)	C4—H4B	0.9800
Mo1—O2	1.9287 (8)	C4—H4C	0.9800
Mo1—O1	1.9788 (8)	C5—C6	1.5137 (15)
Mo1—N1	2.4008 (8)	C5—H5A	0.9900
Mo1—N2	2.4117 (9)	C5—H5B	0.9900
F1—C8	1.3554 (13)	C6—C11	1.3968 (14)
F2—C10	1.3444 (13)	C6—C7	1.4048 (15)
F3—C15	1.3605 (14)	C7—C8	1.3779 (16)
F4—C17	1.3506 (14)	C7—H7	0.9500
O2—C11	1.3494 (12)	C8—C9	1.3835 (18)
O1—C18	1.3494 (12)	C9—C10	1.3767 (16)
N1—C5	1.4894 (13)	C9—H9	0.9500
N1—C1	1.4936 (13)	C10—C11	1.3997 (14)
N1—C12	1.4994 (13)	C12—C13	1.4993 (15)
N2—C4	1.4825 (14)	C12—H12A	0.9900
N2—C2	1.4863 (14)	C12—H12B	0.9900
N2—C3	1.4890 (14)	C13—C18	1.3945 (15)
C1—C2	1.5202 (15)	C13—C14	1.3956 (15)
C1—H1A	0.9900	C14—C15	1.3809 (18)
C1—H1B	0.9900	C14—H14	0.9500
C2—H2A	0.9900	C15—C16	1.377 (2)
C2—H2B	0.9900	C16—C17	1.3835 (16)
C3—H3A	0.9800	C16—H16	0.9500
C3—H3B	0.9800	C17—C18	1.3965 (15)
C3—H3C	0.9800		
			
O3—Mo1—O4	108.54 (4)	H4A—C4—H4C	109.5
O3—Mo1—O2	99.96 (4)	H4B—C4—H4C	109.5
O4—Mo1—O2	95.43 (4)	N1—C5—C6	116.27 (8)
O3—Mo1—O1	98.19 (4)	N1—C5—H5A	108.2
O4—Mo1—O1	93.41 (4)	C6—C5—H5A	108.2
O2—Mo1—O1	156.09 (3)	N1—C5—H5B	108.2
O3—Mo1—N1	160.10 (4)	C6—C5—H5B	108.2
O4—Mo1—N1	91.35 (3)	H5A—C5—H5B	107.4
O2—Mo1—N1	77.83 (3)	C11—C6—C7	119.31 (10)
O1—Mo1—N1	79.79 (3)	C11—C6—C5	123.44 (9)
O3—Mo1—N2	86.27 (4)	C7—C6—C5	117.24 (9)
O4—Mo1—N2	165.19 (3)	C8—C7—C6	119.25 (10)
O2—Mo1—N2	81.21 (3)	C8—C7—H7	120.4
O1—Mo1—N2	84.59 (3)	C6—C7—H7	120.4
N1—Mo1—N2	73.84 (3)	F1—C8—C7	119.02 (11)
C11—O2—Mo1	130.56 (7)	F1—C8—C9	117.80 (11)
C18—O1—Mo1	118.88 (6)	C7—C8—C9	123.17 (10)
C5—N1—C1	110.73 (8)	C10—C9—C8	116.50 (10)
C5—N1—C12	107.20 (8)	C10—C9—H9	121.7
C1—N1—C12	107.89 (8)	C8—C9—H9	121.7
C5—N1—Mo1	108.18 (6)	F2—C10—C9	119.27 (10)
C1—N1—Mo1	107.55 (6)	F2—C10—C11	117.50 (10)
C12—N1—Mo1	115.29 (6)	C9—C10—C11	123.23 (10)
C4—N2—C2	109.15 (9)	O2—C11—C6	124.11 (9)
C4—N2—C3	107.68 (9)	O2—C11—C10	117.37 (9)
C2—N2—C3	109.18 (8)	C6—C11—C10	118.50 (9)
C4—N2—Mo1	109.89 (6)	C13—C12—N1	114.57 (8)
C2—N2—Mo1	111.74 (6)	C13—C12—H12A	108.6
C3—N2—Mo1	109.11 (6)	N1—C12—H12A	108.6
N1—C1—C2	110.46 (8)	C13—C12—H12B	108.6
N1—C1—H1A	109.6	N1—C12—H12B	108.6
C2—C1—H1A	109.6	H12A—C12—H12B	107.6
N1—C1—H1B	109.6	C18—C13—C14	120.78 (10)
C2—C1—H1B	109.6	C18—C13—C12	116.82 (9)
H1A—C1—H1B	108.1	C14—C13—C12	122.11 (10)
N2—C2—C1	111.22 (9)	C15—C14—C13	118.05 (11)
N2—C2—H2A	109.4	C15—C14—H14	121.0
C1—C2—H2A	109.4	C13—C14—H14	121.0
N2—C2—H2B	109.4	F3—C15—C16	118.40 (11)
C1—C2—H2B	109.4	F3—C15—C14	118.20 (12)
H2A—C2—H2B	108.0	C16—C15—C14	123.39 (11)
N2—C3—H3A	109.5	C15—C16—C17	117.20 (11)
N2—C3—H3B	109.5	C15—C16—H16	121.4
H3A—C3—H3B	109.5	C17—C16—H16	121.4
N2—C3—H3C	109.5	F4—C17—C16	119.01 (10)
H3A—C3—H3C	109.5	F4—C17—C18	118.73 (10)
H3B—C3—H3C	109.5	C16—C17—C18	122.26 (11)
N2—C4—H4A	109.5	O1—C18—C13	120.64 (9)
N2—C4—H4B	109.5	O1—C18—C17	121.10 (10)
H4A—C4—H4B	109.5	C13—C18—C17	118.26 (10)
N2—C4—H4C	109.5		
			
C5—N1—C1—C2	168.55 (8)	C9—C10—C11—O2	−179.23 (10)
C12—N1—C1—C2	−74.42 (10)	F2—C10—C11—C6	−178.89 (9)
Mo1—N1—C1—C2	50.54 (9)	C9—C10—C11—C6	2.21 (16)
C4—N2—C2—C1	152.57 (9)	C5—N1—C12—C13	−79.49 (11)
C3—N2—C2—C1	−89.97 (10)	C1—N1—C12—C13	161.21 (9)
Mo1—N2—C2—C1	30.82 (10)	Mo1—N1—C12—C13	41.02 (11)
N1—C1—C2—N2	−55.84 (11)	N1—C12—C13—C18	−57.38 (13)
C1—N1—C5—C6	−61.33 (11)	N1—C12—C13—C14	128.74 (11)
C12—N1—C5—C6	−178.78 (9)	C18—C13—C14—C15	−0.08 (16)
Mo1—N1—C5—C6	56.29 (10)	C12—C13—C14—C15	173.57 (10)
N1—C5—C6—C11	−21.87 (15)	C13—C14—C15—F3	−179.23 (10)
N1—C5—C6—C7	157.75 (9)	C13—C14—C15—C16	1.60 (18)
C11—C6—C7—C8	0.42 (16)	F3—C15—C16—C17	179.98 (11)
C5—C6—C7—C8	−179.21 (10)	C14—C15—C16—C17	−0.85 (18)
C6—C7—C8—F1	−179.79 (10)	C15—C16—C17—F4	179.60 (10)
C6—C7—C8—C9	0.90 (18)	C15—C16—C17—C18	−1.46 (17)
F1—C8—C9—C10	−179.97 (10)	Mo1—O1—C18—C13	68.58 (11)
C7—C8—C9—C10	−0.66 (17)	Mo1—O1—C18—C17	−112.20 (9)
C8—C9—C10—F2	−179.82 (10)	C14—C13—C18—O1	177.19 (10)
C8—C9—C10—C11	−0.93 (17)	C12—C13—C18—O1	3.22 (14)
Mo1—O2—C11—C6	−31.59 (15)	C14—C13—C18—C17	−2.06 (16)
Mo1—O2—C11—C10	149.93 (8)	C12—C13—C18—C17	−176.03 (9)
C7—C6—C11—O2	179.65 (10)	F4—C17—C18—O1	2.58 (15)
C5—C6—C11—O2	−0.74 (16)	C16—C17—C18—O1	−176.36 (10)
C7—C6—C11—C10	−1.89 (15)	F4—C17—C18—C13	−178.18 (9)
C5—C6—C11—C10	177.72 (10)	C16—C17—C18—C13	2.88 (16)
F2—C10—C11—O2	−0.32 (14)		
